# The Preventive Control of Zoonotic Visceral Leishmaniasis: Efficacy and Economic Evaluation

**DOI:** 10.1155/2017/4797051

**Published:** 2017-05-15

**Authors:** Helio Junji Shimozako, Jianhong Wu, Eduardo Massad

**Affiliations:** ^1^Faculty of Medicine, University of São Paulo and LIM 01-HCFMUSP, Avenida Dr. Arnaldo 455, 01246-903 São Paulo, SP, Brazil; ^2^Centre for Disease Modelling, York Institute for Health Research, York University, 4700 Keele Street, Toronto, ON, Canada M3J 1P3; ^3^London School of Hygiene and Tropical Medicine, University of London, London, UK

## Abstract

Zoonotic Visceral Leishmaniasis (ZVL) is one of the world's deadliest and neglected infectious diseases, according to World Health Organization. This disease is one of major human and veterinary medical significance. The sandfly and the reservoir in urban areas remain among the major challenges for the control activities. In this paper, we evaluated five control strategies (positive dog elimination, insecticide impregnated dog collar, dog vaccination, dog treatment, and sandfly population control), considering disease control results and cost-effectiveness. We elaborated a mathematical model based on a set of differential equations in which three populations were represented (human, dog, and sandfly). Humans and dogs were divided into susceptible, latent, clinically ill, and recovery categories. Sandflies were divided into noninfected, infected, and infective. As the main conclusions, the insecticide impregnated dog collar was the strategy that presented the best combination between disease control and cost-effectiveness. But, depending on the population target, the control results and cost-effectiveness of each strategy may differ. More and detailed studies are needed, specially one which optimizes the control considering more than one strategy in activity.

## 1. Introduction

Zoonotic Visceral Leishmaniasis (ZVL) is one of the world deadliest and neglected infectious diseases, according to World Health Organization. This disease is endemic in 80 countries worldwide, in which 90% of all cases occur in Bangladesh, Brazil, India, Nepal, and Sudan. Thus, about 360 million of people are exposed to risk of infection in the world [1–4]. The ZVL is a disease of major human and veterinary medical significance that involves a complex interplay between trypanosomatids protozoan from* Leishmania* complex, arthropod vectors (in Brazil, we find the female sandflies* Lutzomyia longipalpis* and* Lutzomyia cruzi*), environmental influence on vector distribution, small companion animal (dog) reservoir of infection, and susceptible human populations. In American continent,* Leishmania infantum chagasi* is the most important species from* Leishmania* complex.

From the last few years, ZVL has been emerging within nonendemic areas, mostly because of transportation of dogs from endemic areas and climatic changes with the expansion of the geographical range of the sandfly vector. Thus, the effective control will essentially involve interdisciplinary teams of microbiologists, parasitologists, entomologists, ecologists, epidemiologists, immunologists, veterinarians, public health officers, and human physicians [5].

Besides the publication of guidelines of ZVL control and the investments made in general surveillance activities, the sandfly and the reservoir in urban areas remain among the major challenges for the control activities. These challenges are due to (1) the necessity to better understand the vector behavior in urban environment, (2) the operational and logistic difficulties to carry out activities in sufficient time to obtain good results, and (3) the high costs involved in these activities [2, 6].

Usually, health is not analyzed as an economical activity. However, economical analysis in health studies is important for comprehension of health polices dynamics and trends. From those results, it is possible to obtain arguments and support to organize and supervise health polices programs. In short, economic health expresses the universal desire of reaching the best investment, not only in terms of clinical effectiveness, but also in terms of approaching cost-effectiveness about healthcare procedures [7, 8].

Marinho et al. [9] observed that there are few studies that analyzed the economical impact on visceral leishmaniasis considering social and collective approach. In addition, there are several difficulties to develop economical analysis of visceral leishmaniasis transmission due to (I) the interval of time between the intervention and epidemiological impact or/and (II) the difficulty to relate the intervention activities to the resulting impact. Considering those difficulties and still open-questions about ZVL dynamics and impact, the use of mathematical models should become a very interesting alternative of analysis.

Some deterministic models have been published in literature and all of them analyze the dynamic of this disease and make any evaluation of strategies controls. In particular, since 1998 our research group has been working on ZVL modeling. Burattini et al. [10] worked on a mathematical model to visceral leishmaniasis where both humans and dogs were considered source of infection. Later, Ribas et al. [11] developed a model, based on Burattini et al. [10], which was restricted to LVZ and some preventive control strategies were also considered. Newly, an original article was published by Shimozako et al. [12], where they reviewed the model published by Burattini et al. [10] and not only updated some parameters but also provided a more complete mathematical analysis. In this most recent paper, we were able to fit the model to real data from Araçatuba/SP city (Brazil), carrying out a very robust model and results. And, besides those models published by our research team, we also have other researchers who published mathematical models for LVZ, as Zhao et al. [13], in which their model differs from ours by the adopted mathematical structure and the presence of backward bifurcation.

Even though the result from mathematical model indicates epidemiological availability for visceral leishmaniasis control, we should evaluate carefully the practical and economical viability. In this case, regarding public health, the disease control activities should work considering the best cost-effectiveness, since the available resources are limited. We also know that it is important to be aware of the time-response and applicability-practicality conditions. In other words, it is necessary to be careful with investment time and method application relationship and the respective expected result [5, 14–17].

In this work we propose an evaluation of five ZVL control strategies (positive dog elimination, insecticide impregnated dog collar, dog vaccination, dog treatment, and sandfly population control), by mathematical modeling. This mathematical model was based on the previous models published by Burattini et al. [10] and Ribas et al. [11]. We studied the impact of those control strategies on human and dog population by approaching the epidemiological control and cost-effectiveness. Then, we discussed the most efficient control strategies and how they act on visceral leishmaniasis epidemiological chain.

## 2. The Model

We used a mathematical model that is an adaptation of the one proposed by Burattini et al. [10]. In our model, we assume

1. a human and a dog population, with the biological vector transmitting the infection within and between the two populations;
2. those three populations (humans, dogs, and vectors) being constants;
3. both human (indexed as* h*) and dog (indexed as* d*) populations being divided into four categories: susceptible (*x*_*h*_ and* x*_*d*_), infected but without noticeable disease (*l*_*h*_ and* l*_*d*_) (i.e., “latent”), clinically ill (*y*_*h*_ and* y*_*d*_), and recovering immunes (*z*_*h*_ and* z*_*d*_). On the other hand, the vector population is divided into three categories: noninfected, infected but not infective, and infective individuals, denoted as *s*_1_, *s*_2_, and *s*_3_, respectively.

**Figure 1:**
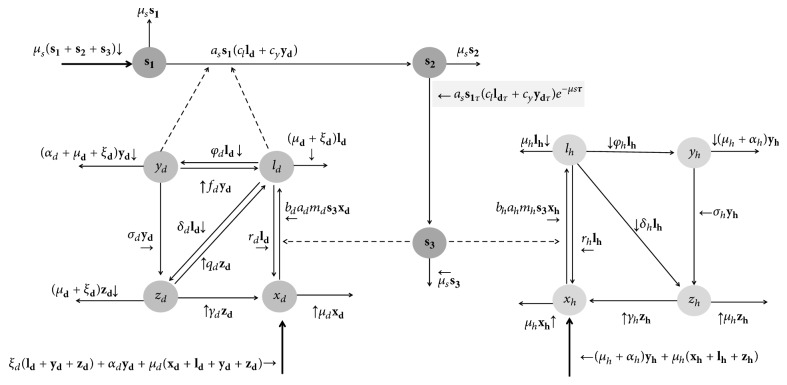
The compartment model and the flowchart. Note that only dogs are source of infection and sandflies transmit the* Leishmania *sp. to both dogs and humans.

The flowchart and compartment model (Figure 1) and the set of differential equations describing the model's dynamics (system (1)) are presented as shown in Figure 1 and are as follows:

(1) xh˙t=μhlht+yht+zht+rhlht+αhyht+γhzht−bhahmhts3txhtlh˙t=bhahmhts3txht−μh+rh+δh+φhlhtyh˙t=φhlht−μh+αh+σhyhtzh˙t=δhlht+σhyht−μh+γhzhtxd˙t=μd+ξdldt+ydt+zdt+rdldt+αdydt+γdzdt−bdadmdts3txdtld˙t=bdadmdts3txdt−μd+rd+δd+φd+ξdldtyd˙t=φdldt−μd+αd+σd+ξdydtzd˙t=δdldt+σdydt−μd+γd+ξdzdts1˙t=μss2t+s3t−asclldt+cyydts1ts2˙t=asclldt+cyydts1t−μss2t−asclldt−τ+cyydt−τs1t−τe−μsτs3˙t=asclldt−τ+cyydt−τs1t−τe−μsτ−μss3t.

The definition, biological meaning, and values of each of parameter are described in Table 1.

A brief description of system (1) should clarify their meaning.

Let *S* be the total number of sandflies. The number of bites inflicted in the human host population in an infinitesimal time interval *dt* is *a*_*h*_*S*(*t*)*dt*, where* a*_*h*_ is the biting rate on humans. The number of bites inflicted by infected flies is *a*_*h*_*S*(*t*)*dtS*_3_(*t*)/*S*(*t*) = *a*_*h*_*S*(*t*)*dts*_3_(*t*), where *S*_3_(*t*) is the number of infected flies.

Let now *X*_*h*_(*t*)_*h*_ be the total number of susceptible individuals in the human population. In an infinitesimal time interval *dt*, *X*_*h*_(*t*) varies as follows:

- The infected flies are able to bite on any category of human population. Thus, only a fraction of the infected bites are on uninfected individuals: *a*_*h*_*S*(*t*)*dts*_3_(*t*)*x*_*h*_(*t*), where *x*_*h*_(*t*) is the fraction of uninfected humans. But, a fraction* b*_*h*_ of *a*_*h*_*S*(*t*)*dts*_3_(*t*)*x*_*h*_(*t*) becomes latent, so *X*_*h*_ diminishes by *b*_*h*_*a*_*h*_*S*(*t*)*dts*_3_(*t*)*x*_*h*_(*t*).
- Simultaneously, *r*_*h*_*L*_*h*_(*t*)*dt* + *γ*_*h*_*Z*_*h*_(*t*)*dt* individuals, latent and immune, revert to the susceptible condition, and *μ*_*h*_*X*_*h*_(*t*)*dt* die by natural causes other than the disease.
- We must add an entrance term, due to natality, which we choose to be *α*_*h*_*Y*_*h*_(*t*)*dt* + *μ*_*h*_*N*_*h*_(*t*)*dt*, where *α*_*h*_ is the disease-induced mortality rate, *Y*_*h*_(*t*) is the number of infected humans (clinically ill humans), and *N*_*h*_(*t*) is the total number of humans needed to maintain a constant population (where *N*_*h*_(*t*) = *X*_*h*_(*t*) + *L*_*h*_(*t*) + *Y*_*h*_(*t*) + *Z*_*h*_(*t*), with *L*_*h*_(*t*) as the number of latent humans and *Z*_*h*_(*t*) as the number of recovering humans).

Thus we have

(2) dXh=αhYhtdt+μhNhtdt−bhahStdts3txht+rhLhtdt+γhZhtdt−μhXhtdt.

Dividing this equation by *N*_*h*_(*t*)*dt* and calling *S*(*t*)/*N*_*h*_(*t*) = *m*_*h*_, we get the first equation of system (1).

Observe that *m*_*h*_ is a time-dependent function: *m*_*h*_(*t*). This expression is the simplest way to simulate the changes on sandfly population size dynamics between 1999 and 2015.

We can apply the same process in order to obtain the equation for the dynamic of susceptible dogs (*x*_*d*_). However, observe from Table 1 that the sandfly : dog ratio depends on the sandfly : human ratio and on the human : dog ratio: *m*_*d*_ = *m*_*h*_(*t*) × *w*_*dh*_. Although all the populations are constant, if we consider the real number of individuals, we expect more humans than dogs. Thus, if the sandfly population is constant, we have different values for *m*_*d*_ and *m*_*h*_.

The last three equations of system (1) refer to the flies. When infected, a fly remains in a latent stage for a period of time *τ*. This time corresponds to the extrinsic incubation period of the parasite inside the vector fly. Numerically it lasts for about half the life expectancy of the flies.

Let *S*_1_ be the number of susceptible flies. In an infinitesimal period of time *dt*, (*a*_*S*_(*L*_*d*_(*t*) + *Y*_*d*_(*t*)/*N*_*d*_(*t*))*dt*)*S*_1_(*t*) bites due to uninfected flies occur on latent and infected dogs (humans are not considered to be infective for flies; see Tesh [18]). A fraction, *c*_*l*_ and *c*_*y*_, of the flies (that bites latent and clinically ill dogs, resp.) becomes latently infected as a result. Therefore, we have

(3) dS1t=μsS2t+S3tdt−aSclldt+cyydtS1tdt.

Dividing by *S*(*t*) = *S*_1_(*t*) + *S*_2_(*t*) + *S*_3_(*t*) and by *dt*, we get the equation for noninfected sandflies (*s*_1_(*t*)).

Although this is a brief but detailed description of the noninfected categories equations (i.e., *x*_*h*_, *x*_*d*_, and *s*_1_), we can note that each term of our system equation has a biological meaning. The meaning of each term depends on the respective parameters that set them (e.g., *δ*_*d*_*l*_*d*_(*t*) + *σ*_*d*_ means the amount of latent dogs that develop immunity per day).

## 3. The Number of Clinically Ill Humans and Reported Cases

In Brazil, ZVL is a notifiable disease [17, 34]. Thus, we can assume the following:

- An infected human should look for medical treatment when he/she will become clinically ill (*y*_*h*_).
- Only a fraction of those humans that are clinically ill will be reported to sanitary authorities. The remaining fraction (I) will not look for medical help, even if the clinical symptoms and signs appear or (II) will not be correctly reported in the hospitals.

Now, let us see again the equation for *y*_*h*_(*t*) in system (1):

(4) $$\begin{array}{c} \dot{y_{h}}\left(\;t\;\right)=\varphi_{h}l_{h}\left(\;t\;\right)-\left(\;\mu_{h}+\alpha_{h}+\sigma_{h}\;\right)y_{h}\left(\;t\;\right). \end{array}$$

The term *φ*_*h*_*l*_*h*_(*t*) in (4) means the rate of latent humans who become clinically ill per day. Thus, in order to calculate the total of humans that become clinically ill along an interval of time, we have

(5) $$\begin{array}{c} T_{y_{h}}\left(\;t_{f}\;\right)=\varphi_{h}\overset{t_{f}}{\underset{t_{0}}{\int }}l_{h}\left(\;t\;\right)dt, \end{array}$$

where *T*_*y*_*h*__(*t*_*f*_) is the total of humans that become clinically ill from an initial moment, *t*_0_, to a final one, *t*_*f*_.

Now, let us consider that, per day, the number *φ*_*h*_*l*_*h*_(*t*) of humans is eligible to look for medical help. However, only a fraction (1 − *η*_*h*_) of those clinically ill humans will be correctly notified to sanitary authorities, where *η*_*h*_ = 0.705 means the proportion of unreported cases [35]. Therefore, the daily rate of reported human cases Rep(*t*) is defined by

(6) $$\begin{array}{c} Rep\left(\;t\;\right)={\left({1-\eta }_{h}\right)\varphi }_{h}l_{h}\left(\;t\;\right). \end{array}$$

The Centre of Epidemiological Surveillance of São Paulo State (CES-SP) [36] is the institution that administrates the data about ZVL in São Paulo State. In order to validate our model, we decided to use the data of human reported cases from the municipality of Araçatuba (São Paulo State, Brazil) as reference, because it is an endemic city for this disease. Those data are presented in Table 3 and are available on CES-SP website [36].

Note that we have the total of reported cases per year. Thus, since our time scale is* day*, we estimated an average of human reported cases per day for each year (dividing the total from each year by 365). Finally, we also have to consider that our model works with normalized population (all three populations are constant). Thus, as a last step, we have to divide each rate of human reported cases per day by the official population size of Araçatuba municipality. The population size of Araçatuba municipality is available on Brazilian Institute of Geography and Statistics website [22]

In order to fit and compare our results to real data, we also calculated a normalized average of reported cases per day from every 365 days of simulation. This simulation was run considering 60 years and the obtained curve was fitted by simple handling along the time-axis (e.g., we could assume the initial day *t*_0_ = 1 as the first day of 1970 or 1980, depending on how best the simulated curve fits on the real data). Thus, we could obtain the yearly average of reported human cases per day and compare it to the real yearly average provided by CES-SP [36] (Table 3).

## 4. Fitting the Human : Sandflies Ratio (*m*_*h*_(*t*))

Among all used parameters for this work, the sandfly/human ratio is one of the most challenging to be estimated. Although we had found some field studies that tried to estimate sandfly population size and other demographic characteristics [37], we did not find any study regarding this ratio for Araçatuba city. Therefore, in our simulation, we decided to fit this ratio according to real data of human cases. Since we are studying visceral leishmaniasis dynamics, it is necessary that the disease is persistent in the population. Considering this condition, we assumed the condition *ℛ*_0_ > 1 and estimated the minimum value for *m*_*h*_(*t*) (calculation is not shown, but we followed the method described by van den Driessche and Watmough [38]).

The real data provided in Table 3 suggests that the incidence was not constant along those years in which the data was collected (1999 to 2015). One reasonable hypothesis is the climate changes that have been occurring for the last years [39]. Thus, since the sandfly population dynamics depend on climate and geographical conditions, we can include this idea in our model by fitting *m*_*h*_(*t*) as time-function. It is not the scope of this paper to model the sandfly population dynamics according to climatic and geographic variations. Therefore, we will assume that a simple function for *m*_*h*_(*t*), which can fit the simulation data to the real data, should include those climatic and geographic variabilities.

Let us consider the following function for *m*_*h*_(*t*):

(7) mht=mh0+te−L+t/K1K1A+Bsin⁡2πtTlimt→+∞⁡mht=mh0.

The parameter values for (7) are in Table 4. Biologically, we can suppose that sandfly population reaches stability and oscillations decrease over time. Thus, note that for *t* → +*∞* we have *m*_*h*_(*t*) trending to *m*_*h*0_.

## 5. Modeling the Dynamic of Control Strategies

System (1) models the disease dynamics over time, considering humans, dogs, and sandfly population. In order to evaluate the effect of preventive controls, we have to introduce new terms that indicate each of those methods. Since our focus is preventive control method, the target populations are dogs and sandflies.

In the following sections we present the inclusion of those new terms on system (1). We consider the parameters from Araçatuba municipality for simulation of those methods.

Each of the five control strategies considered in this work acts in a specific point of the ZVL dynamics. Because of this, it becomes clearer if we redescribe our model for each strategy separately. Therefore, we simulated 6 sceneries (one without control strategies and one for each strategy) and, for each evaluated strategy, we counted the number of individuals (dog or houses) that were controlled.

The estimation of control strategy rates is presented apart in the following sections.

### 5.1. Elimination of Positive Dogs

(8) xd˙t=μd+ξd+ξ′dldt+ydt+zdt+rdldt+αdydt+γdzdt−bdadmdts3txdtld˙t=bdadmdts3txdt−μd+rd+δd+φd+ξd+ξ′dldtyd˙t=φdldt−μd+αd+σd+ξd+ξ′dydtzd˙t=δdldt+σdydt−μd+γd+ξd+ξ′dzdt.

The elimination of positive dogs has already been indicated as *ξ*_*d*_ in system (1), in the equations for dog population, and in Table 1. In this case, we suppose that this elimination rate is in accordance with the average produced by epidemiological surveillance system of Araçatuba [31]. In other words, *ξ*_*d*_ means the usual dog elimination rate (i.e., the dog elimination provided by health authorities in a common routine). In addition, since the official diagnosis method is serology, we assume any dog that is indicated as having antibody against* Leishmania *parasite as disease positive.

Note from Figure 1 that dog population is considered constant in our model. As a result, if the dog mortality is intensified due to elimination of positive ones (i.e., there is an extra/additional elimination rate by *ξ*′_*d*_, e.g., if the health services receive better working conditions and if they are supplied by more materials), it induces an increase of dog population renewing. This renewing makes sense, since, ecologically, an eliminated dog allows a new one to replace it. In addition, as the official diagnostic techniques are based on serology, only susceptible dogs *x*_*d*_ are not eligible to be eliminated. We adopted this idea because we considered the latent (*l*_*d*_), clinically ill (*y*_*d*_), and recovering (*z*_*d*_) dogs had contracted the* Leishmania* antigen in any moment of its life. Therefore, they are eligible to be positive for diagnostic test.

### 5.2. Deltamethrin 4% Impregnated Dog Collar

Theoretically, the deltamethrin 4% impregnated dog collar could be applied in any dog. Therefore, we can assume that all of the four classes of dog in our model are eligible to use it and we adopted *θ*_*d*_ as the rate of dogs using collar per day. In this case, we indicated by *C* the categories of dogs that use collar (susceptible dogs using collar *x*_*d*_^*C*^, latent dogs using collar *l*_*d*_^*C*^, clinically ill dogs using collar *y*_*d*_^*C*^, and recovering dog using collar *z*_*d*_^*C*^) from those that do not use it. Basically, once a dog has this collar, it becomes protected from sandfly biting. If there is no contact between them, there will not be parasite transmission (either from infected dog to noninfected sandfly or from infective sandfly to susceptible dog).

Also, let us assume that those collars are available for inhabitants at local health centers. Thus, we suppose that owners would actively go to health center and acquire the collar for each dog they have. Since we consider that all preventive activities are supported by health policies, we can consider that the owner acquires the collar with no charge. If we imagine this simple hypothesis, we conclude that the only additional cost to the health policies is the purchasing of the collar.

**Figure 2:**
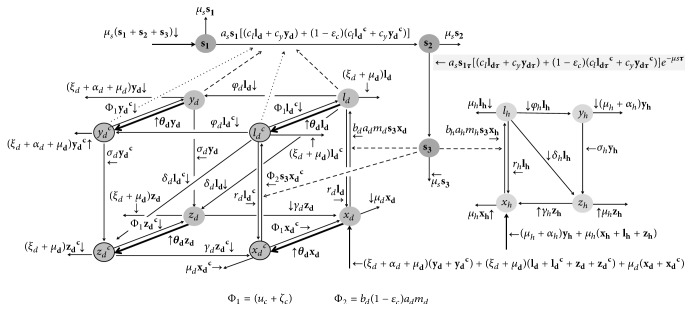
The compartment model and the flowchart, when the vaccination is introduced as preventive strategy control. Note that the dynamics for human population have not changed.

Figure 2 refers to the flowchart considering the inclusion of deltamethrin 4% impregnated dog collar. Next, we have system (9), in which we included the collar-classes, and Table 5 where we describe the additional parameters for this control.

Note from Figure 2 that once the collar is fitted, there is a loss rate *ζ*_*c*_ and a decrease of insecticide effect rate *u*_*c*_. Also, according to Halbig et al. [42], the efficacy of the collar is around 80%. Therefore, we considered that a proportion *ε*_*c*_ of those dogs using collar is protected.

(9) xd˙t=Bt+uc+ζcxdCt+rdldt+γdzdt−θd+bdadmds3txdtld˙t=uc+ζcldCt+bdadmds3txdt−μd+rd+δd+φd+ξd+θdldtyd˙t=uc+ζcydCt+φdldt−μd+αd+σd+ξd+θdydtzd˙t=uc+ζczdCt+δdldt+σdydt−μd+γd+ξd+θdzdtBt=μd1−xdt+ξd1−xdt−xdCt+αdydt+ydCtxdC˙t=θdxdt−μd+uc+ζc+1−εcbdadmds3txdCt+rdldCt+γdzdCtldC˙t=θdldt+1−εcbdadmds3txdCt−μd+rd+δd+φd+ξd+uc+ζcldCtydC˙t=θdydt+φdldCt−μd+αd+σd+ζc+uc+ξdydCtzdC˙t=θdzdt+δdldCt+σdydCt−μd+γd+ζc+uc+ξdzdCts1˙t=μss2t+s3t−asIdt+IdCts1ts2˙t=asIdt+IdCts1t−μss2t−asIdt−τ+IdCt−τs1t−τe−μsτs3˙t=asIdt−τ+IdCt−τs1t−τe−μsτ−μss3tIdt=clldt+cyydtIdCt=1−εcclldCt+cyydCt.

### 5.3. Dog Vaccination

Biologically, the vaccination would be effective only in susceptible dogs *x*_*d*_, avoiding them to become infected by infective sandfly bites. Thus, if the vaccine distribution was only for susceptible dogs, it would be necessary to submit several dogs to diagnostic procedure. However, in practical terms, this is not feasible. Therefore, we suppose that all dogs are eligible to be vaccinated and this category of vaccinated dogs is indicated by *v*_*d*_ (lowercase “*v*”).

In our model, we considered that leishmaniasis vaccination would be offered together with rabies vaccine. In other words, we suppose that the rabies vaccination campaign would distribute not only rabies vaccines but also leishmaniasis vaccine. Since the rabies vaccination campaign has been already included in the annual municipality budget, the minimum additional cost to operation of vaccination as control strategy would be only the leishmaniasis vaccine purchasing. This is an idea similar to the one adopted to dog collar. However, in this model we are considering only the leishmaniasis vaccination rate *υ*_*d*_ (lowercase “ipsilon”) and its respective impact as control activity.

**Figure 3:**
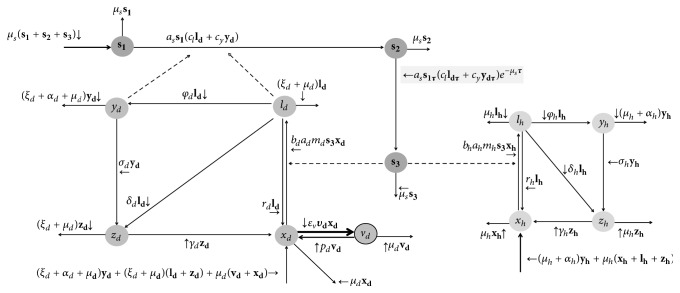
The compartment model and the flowchart, when the vaccination is introduced as preventive strategy control. Note that the dynamics for human and sandfly populations have not changed.

Figure 3 refers to the flowchart considering the inclusion of leishmaniasis vaccination. Next, we have system (10), in which we included the vaccinated dog compartment, and Table 6 where we describe the additional parameters for this control.

Note from Figure 3 that once the dog is vaccinated, there is a loss of immunity rate *p*_*c*_ [43]. Also, according to Fernandes et al. [44], the efficacy of leishmaniasis vaccination is around 96.4%. Therefore, we considered that a proportion *ε*_*v*_ of these vaccinated dogs against leishmaniasis is immunized.

(10) xd˙t=Bt+rdldt+γdzdt+pdvdt−bdadmdts3t+εvυdxdtld˙t=bdadmdts3txdt−μd+rd+δd+φd+ξdldtyd˙t=φdldt−μd+αd+σd+ξdydtzd˙t=δdldt+σdydt−μd+γd+ξdzdtvd˙t=εvυdxdt−pd+μd+ξdvdtBt=μd+ξd1−xdt+αdydt.

### 5.4. Dog Treatment

In this control strategy, the objective is reducing the number of infected dogs, which works as source of infection. However, the probability of treating a latent dog is quite null, since this category of dog is visually healthy. Thus, we assume that only dogs that present clinical signs and/or symptoms are eligible to be treated and the dog treatment rate is indicated as *ω*_*d*_.

We will consider the treatment protocol described by Miró et al. [45], which was composed by meglumine antimoniate plus allopurinol. In this work, the authors found a proportion of dogs that healed but still continued to be infected. In other words, once a dog is treated, there is a probability of a dog eliminating the parasitemia or not.

Furthermore, we also consider that the dog treatment would be offered by public health policies. Therefore, if the public health services have already included veterinarians in the staff, the minimum additional cost would be the acquisition of the medicine (meglumine antimoniate and allopurinol) and hospital material (e.g., syringes and needles).

**Figure 4:**
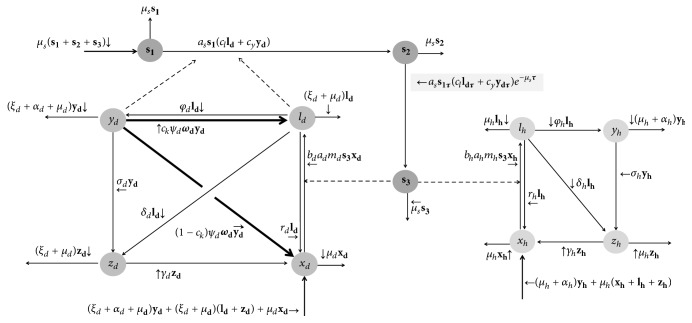
The compartment model and the flowchart, when the dog treatment is introduced as preventive strategy control. Note that the dynamics for human and sandfly populations have not changed.

Figure 4 refers to the flowchart considering the inclusion of dog treatment. Then, we have system (11), in which we included the treated dogs flux (from clinically ill to susceptible or to latent), and Table 7 where we describe the additional parameters for this control.

Note from Figure 4 that once the dog is treated, there is a probability to be recovered, but without parasitemia elimination. We adopt *c*_*k*_ as a proportion of dogs that obtain only clinical recovery but are still infected [45]. Also, once the treatment started, we assumed that any dog gives up on the treatment process over time (i.e., the proportion of dogs that receive the complete treatment is *ψ*_*d*_ = 1).

(11) xd˙t=Bt+1−ckψdωdydt+rdldt+γdzdt−bdadmdts3txdtld˙t=ckψdωdydt+bdadmdts3txdt−μd+rd+δd+φd+ξdldtyd˙t=φdldt−μd+αd+σd+ξd+ψdωdydtzd˙t=δdldt+σdydt−μd+γd+ξdzdtBt=μd+ξd1−xdt+αdydt.

### 5.5. Sandfly Population Control

The activities of sandfly population control focus on two approaches, both of them on the environment. First, according to Brazilian Ministry of Health [17], the sandfly population control includes a chemical control (spraying of insecticide on the houses) and a land clearing (that reduces the sandfly carry capacity). In order to simplify our study, we just considered that those both approaches included in the sandfly population control result in an increase of sandfly mortality rate, *ξ*_*s*_. On the other hand, it is unfeasible to organize a sandfly control considering the sandfly mortality rate, as “eliminated sandfly/day” (i.e., working in function of the amount *ξ*_*s*_ × *S*). Because of this, we considered as sandfly control rate the dimension of “treated houses/(sandfly × day)”: *ξ*_*c*_. Therefore, once the number of treated houses to be treated per day and per sandfly is determined, we can easily find the additional sandfly mortality rate:

(12) $$\begin{array}{c} \xi_{s}=\xi_{c}\times w_{hc}\times m_{h0}, \end{array}$$

where *w*_*hc*_ means the average human/house and *m*_*h*0_ is the ratio sandfly/human.

It would be very complex to estimate the sandfly population control budget, but for this model we considered the economical evaluation presented by Camargo-Neves [31] (Table 8).

**Figure 5:**
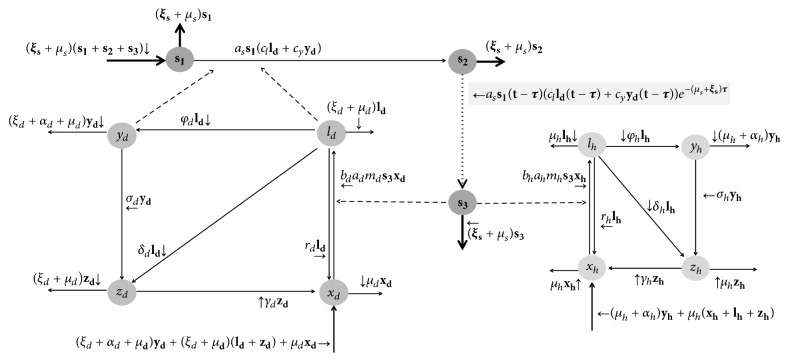
The compartment model and the flowchart, when the sandfly population control is introduced as preventive strategy control. Note that the dynamics for human and dog populations have not changed.

Figure 5 refers to the flowchart considering the inclusion of sandfly population control. Then, we have system (13), in which we presented the additional sandfly mortality rate *ξ*_*s*_ = *ξ*_*c*_*w*_*hc*_*m*_*h*0_.

In a proportional approach, note from Figure 5 that sandfly population is considered constant in our model (we remember that our three populations in our model are normalized). As a result, the proportional increase of its mortality rate induces an increase of population renewing at the same proportion. This acceleration of population renewing refers to the conception of carry capacity. Here, carry capacity means the maximum population size of biological species in an environment. Thus, whenever the sandfly population is under the carry capacity, it will tend to increase until it becomes fitted to it. Also, the opposite occurs if it is over the carry capacity (the population will decrease until its size fits the carry capacity). Finally, as our model considers the sandfly population proportionally constant, it means that when sandflies die, the population will decrease and it will be under the maximum size allowed by carry capacity. As a consequence, the population will increase by recruitment of new individuals (mathematically, this is the entrance term *μ*_*s*_(*S*_2_(*t*) + *S*_3_(*t*))). Therefore, in short, we conclude that if the sandfly mortality rate increases, the sandfly population renewing rate will also increase.

According to Burattini et al. [10], the acceleration of the sandfly population renewing (or, in other words, the decrease of life expectancy of sandfly population) affects directly the LVZ dynamics, since the infected sandfly *s*_2_ is also eliminated in a shorter time. As a consequence, the parasite* Leishmania s*_2_ will not have time enough to complete its development inside the sandfly and the proportion of infective *s*_3_ will also naturally decrease.

(13) s1˙t=μs+ξcwhcmh0s2t+s3t−asIdts1ts2˙t=asIdts1t−μs+ξcwhcmh0s2t−asIdt−τs1t−τe−μs+ξcwhcmh0τs3˙t=asIdt−τs1t−τe−μs+ξcwhcmh0τ−μs+ξcwhcmh0s3tIdt=clldt+cyydt.

## 6. The Estimated Costs and Calculation of Control Strategy Rates

It is very important to consider not only the result of the control strategy at the light of epidemiological approach but also the economical one too. Therefore, since the number of controlled elements is in dimension of elements/day, the estimation of the cost/elements would provide us with the estimated cost per day (i.e., cost/individual × individual/day = cost/day). Here we suppressed the cost calculation of each method, but we indicated the references from where we preceded our estimations. Table 8 summarizes those costs.

Usually, the operating of preventive control strategies is limited by logistic and financial resources. Therefore, in order to estimate the preventive control rates, firstly it is necessary to estimate those restrictions.

First, considering the data from Table 3. From “Human reported cases per year” column we estimated the year average, which is 20.18 human cases/year. Then, from Table 8, the estimated cost for human treatment is around 397.25 USD/human [46]. Therefore, per year, the average expanses with human treatment are around 20.18 × 397.25 = 8015 USD/year. If we consider the costs per day, we have around 22 USD/day. For simplicity, we will consider that this value includes not only financial aspects but also logistic one.

Now, let us suppose that instead of this 22 USD/day that is invested on patient treatments, it would be invested on preventive control strategies. However, we should consider that this 22 USD/day is invested on the prevention of the whole dog population or houses. If we consider the human : dog ratio for Araçatuba/SP city of 10/1.8 human/dog [29] and the human : house ratio of 3 humans/house [22], the estimation of dog population and the number of houses for 2016 is around 34889 dogs and 64609 houses. Then, the invested cost per dog is estimated as 22/34889 = 6.29 × 10^−4^ USD/(dog × day) and, considering houses, 22/64609 = 3.40 × 10^−4^ USD/(house × day). Since we obtained the estimated costs for each control strategy (Table 8), it is possible to estimate the maximum rate of each control. As example, if the cost for elimination of one positive dogs is 170.71 USD/dog, the maximum rate of elimination dog per day would be 6.29 × 10^−4^/170.71 = 3.69 × 10^−6^/day. In the same way, we just repeated the calculation process and estimated the maximum rate of each control strategy, but in the case of vector control we used the estimated number of houses instead of estimated dog population. All estimated control rates are in Table 8.

It is important to present a special consideration about *ξ*_*c*_ dimension. According to (12), *ξ*_*c*_ dimension is “houses/(sandfly × day).” Since the dimension of estimated investment cost per house is “USD/(house × day),” we concluded that the cost estimated of sandfly population control presents the dimension “USD × sandfly/(house)^2^.” This dimension can be splitted as “(USD/house) × (sandfly/house).” Thus, we can observe that the cost of sandfly population control depends on density sandfly/house. The higher this density sandfly/house is, the more expensive the cost becomes. Therefore, we considered the sandfly population control average cost as 23.24 USD × sandfly/(house)^2^.

## 7. The Impact of Control Strategies on Total of Saved Humans

According to Table 2, we accessed official data of Araçatuba municipality from 1999 to 2015. Later, from those data, we were able to fit the model from system (1) by observing the resulting curve from reported human cases in (4) from fitting *m*_*h*_(*t*) in (7).

Once we have the model from system (1) defined and calibrated, we are able to evaluate the dynamics of each control strategy and compare them with the no-control strategy scenery.

**Figure 6:**
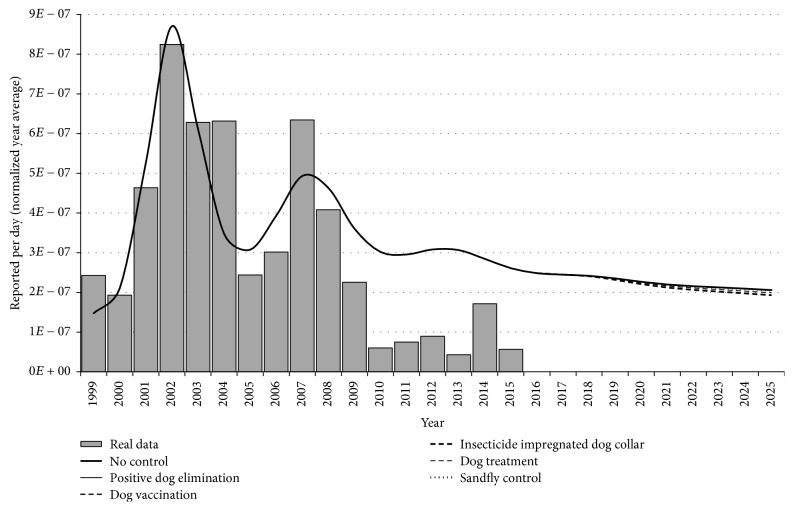
Total disease dynamics and plotting of official data over time. The control strategies were supposed to be introduced from 2018. Observe that the prediction of reported human cases from 2018 for each control strategy is quite close and, therefore, in this scale the curves are overlapped (see Figure 7 for a larger scale).

**Figure 7:**
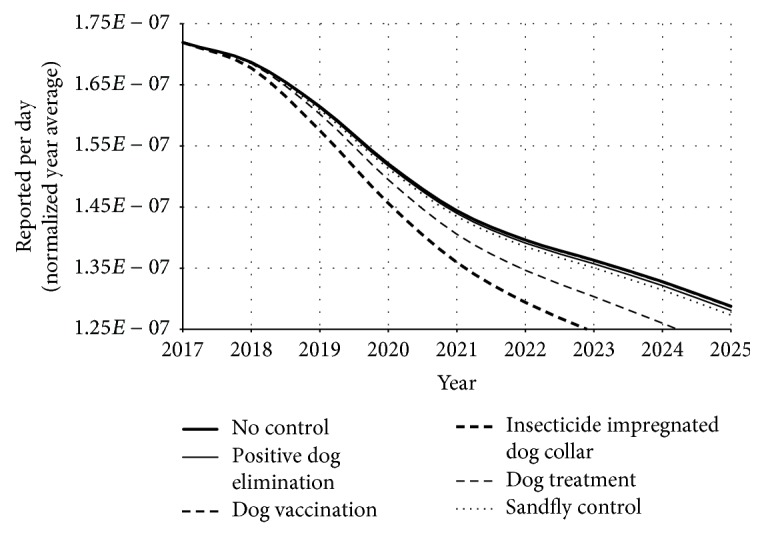
Disease dynamics considering the introduction of control strategies. Here we present a larger scale of the vertical and horizontal axis in order to provide a better observation of the curves. Note that the insecticide impregnated dog collar is the strategy that generates lower reducing of reported human cases. On the other hand, the dog treatment curve is overlapped with the no-control curve. Therefore, it is the strategy that presented the worst result in terms of reported human cases reduction.

First, we considered the numerical simulation of system (1) from 1999 to 2025. Since we are interested in understanding the dynamics of the disease over time in an as real as possible behavior, we present Figure 6 with bars that indicate the official data. However, considering the prediction evaluation of the control strategies, we assumed in our simulation that those control strategies would start to be operated in 2018. Therefore, we observe in Figure 6 the numerical simulation and the prediction result if we consider the introduction of those strategies starting from 2018 (for a better view, see Figure 7).

Once we observed the control strategy dynamics in terms of reported human cases, it is very useful to estimate the quantity of people that avoided the infection. Just for simplification, in this text we refer to those people as “saved” human.

Since we developed a computational simulation, we had the control of sceneries. Therefore, in order to evaluate the impact of each control strategy, we compared the simulation results between introduced control strategy and no-control simulations. It is important to remember that in all simulations we computed the real total of clinically ill humans, according to (14). However, if we calculate the difference between the no-control simulation and introduced control simulation, we have the quantity of humans that were prevented to become clinically ill (the saved one).

**Figure 8:**
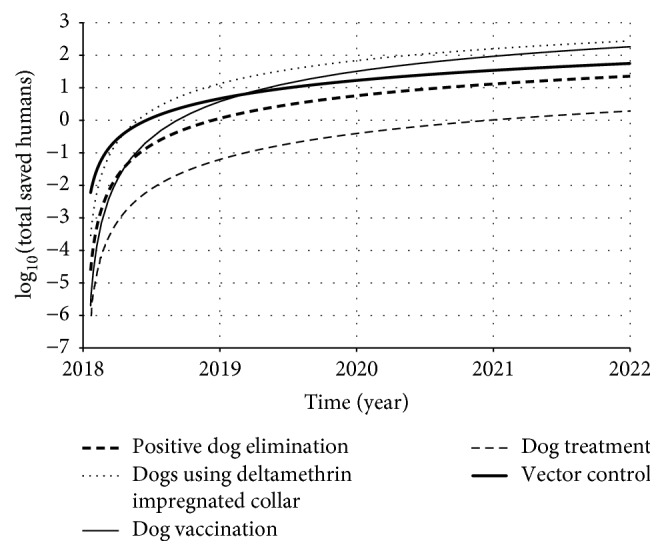
Total of saved humans over time, according to each strategy. Observe that using of deltamethrin impregnated collar and vaccination were the strategies which saved more humans. On the other hand, the dog treatment saved around hundred times fewer individuals, if compared to those two best strategies. Since those curves exponentially grow up, we used a log-scale in vertical axis.

(14) TsavediTyhno-controltf−Tyhitf=φh∫t0tflhno-controlt−lhitdt,

where *𝒯*_saved_^*i*^ is the total of saved humans until time *t*_*f*_ and *i* is the correspondent control strategy.  Figure 8 represents the result of those totals of saved humans for each control strategy.

According to Figure 8, the dog treatment was the strategy that presented the lower number of saved people. It makes some sense, since the dog treatment does not eliminate the parasitemia status. Therefore even if an infected dog is treated, it may still continue being source of infection. On the other hand, the insecticide impregnated dog collar and dog vaccination were the strategies that most saved humans. Those two strategies reduce the amount of exposed susceptible individuals to infective sandfly biting. As a consequence, the proportion of infected humans decreases. However, although this interpretation is correct, we did not consider the restriction of resources, as financial, material, or human support. In the next section, we will include our observations about this.

## 8. Number of Controlled Elements and the Estimation of Total Cost

According to Table 8, each control strategy has a cost per controlled element (dog or house). Therefore, it is essential to understand how to find the equilibrium between the disease control and the available resources (material and/or financial).

In general idea, to count the controlled elements it is necessary to sum the amount of controlled elements per day over an interval of time: total of controlled elements = controlled elements/day × interval of time (days).

From total of controlled elements it is simple to estimate the invested total. Here, we are interested to compare the cost of the control strategies with the cost of human treatment. Therefore, if the cost of each strategy per element has already been normalized in terms of the human treatment cost, we are able to estimate the total cost as total cost = total of controlled elements × cost (normalized by human patient cost)/element.

**Figure 9:**
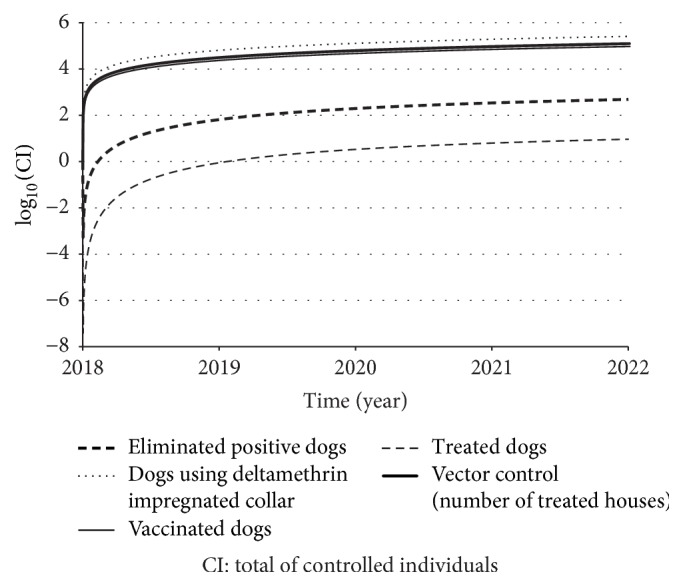
Total of controlled individuals (dogs or houses) over time, according to each strategy. Note that the dynamics of total number of treated houses for vector control and the number of vaccinated dogs are very similar. Since those curves exponentially grow up, we used a log-scale in vertical axis.

**Figure 10:**
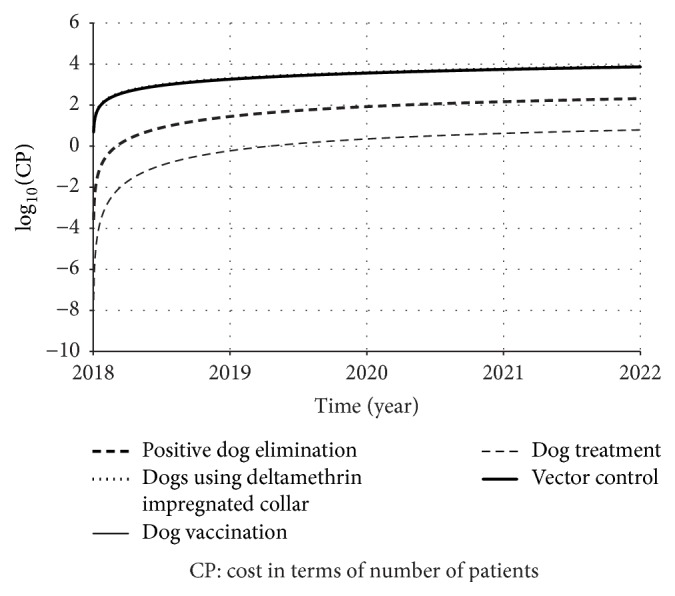
Total cost of each strategy over time, normalized by cost of human treatment. Note that three curves are overlapped: vector control, dog vaccination, and dog using deltamethrin impregnated collar. Since those curves exponentially grow up, we used a log-scale in vertical axis.

Table 9 presents the expressions that calculate the total of controlled elements and the total cost of each strategy. Figures 9 and 10 present, respectively, the dynamics of total of controlled dogs or houses and the total cost normalized by human patient cost.

From Figures 9 and 10, it is possible to observe a similarity and correspondence between the curve responses. In terms of costs, vector control, dog vaccination, and dog collar are very close to each other. But, the difference is related to the number of controlled elements, in which we found that there were more dogs with collar than vaccinated dogs or treated houses. Also, although dog treatment and dog elimination presented reduced costs, they also controlled fewer elements too.

## 9. Calculation of *ℛ*_0_ as Function of Each Preventive Control and Evaluation of *ℛ* Dynamics

For each evaluation, we calculated the respective *ℛ*_0_ (Basic Reproduction Number) in function of the preventive control method. The Basic Reproduction Number indicates the quantity of infected individuals generated from one infective individual, when introduced in a population in disease-free equilibrium state [48]. We assumed that *ℛ*_0_ is calculated when the time *t* is high enough, where lim_*t*→+*∞*_⁡*m*_*h*_(*t*) = *m*_*h*0_. As stated before, the full calculations are not described in this work, but we adopted the review published by van den Driessche and Watmough [38]. Once *ℛ*_0_ is calculated, we calculated the respective *ℛ*(*t*) (effective reproduction number) and investigated which one of the 5 control strategies (elimination of positive dogs *ξ*′_*d*_, use of deltamethrin 4% impregnated dog collar *θ*_*d*_, dog treatment with allopurinol and meglumine antimoniate *ω*_*d*_, dog vaccination *υ*_*d*_, and sandfly population control *ξ*_*c*_) makes *ℛ*(*t*) converge fastest to a value lower than 1.

Table 10 summarizes the *ℛ*_0_ expressions for each control strategy.

The conception of *ℛ*_0_ is restricted on population's disease-free equilibrium state, since the mathematical approach that defines it considers the system in equilibrium states. Usually, a dynamic system has two classes of equilibrium states: a trivial and nontrivial state. If our dynamics system is a disease dynamics one, the trivial equilibrium is this disease-free equilibrium state and it is considering this equilibrium in which *ℛ*_0_ is calculated.

However, it is natural that there is generation of infected individuals immediately after the transmission has started. In this case, it is important to consider the susceptible individual dynamics. Therefore, the number of infected individuals generated from an infective one depends on the remaining susceptible individuals in the population:

(15) $$\begin{array}{c} R\left(\;t\;\right)=R_{0}\times x\left(\;t\;\right), \end{array}$$

where *x*(*t*) is the proportion of susceptible individuals in population. Theoretically, we have two host populations for your model: humans and dogs. From those two populations, we have three classes of susceptibles: *x*_*h*_(*t*), *x*_*d*_(*t*), and (1 − *ε*_*c*_)*x*_*d*_^*C*^(*t*) (since the collar has a proportion of efficacy). Therefore, we estimated *ℛ*(*t*) as

**Figure 11:**
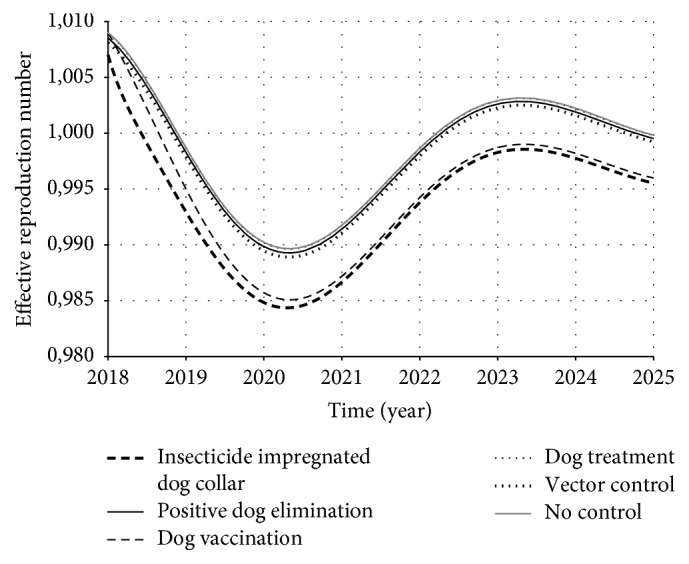
*ℛ*(*t*) dynamics for dog population over time. The control strategies were supposed to be introduced from 2018. Observe that insecticide impregnated dog collar and dog vaccination were the strategies that most reduced *ℛ*(*t*). Note also that dog treatment presented the lowest impact and, therefore, its curve is overlapped with no-control curve.

**Figure 12:**
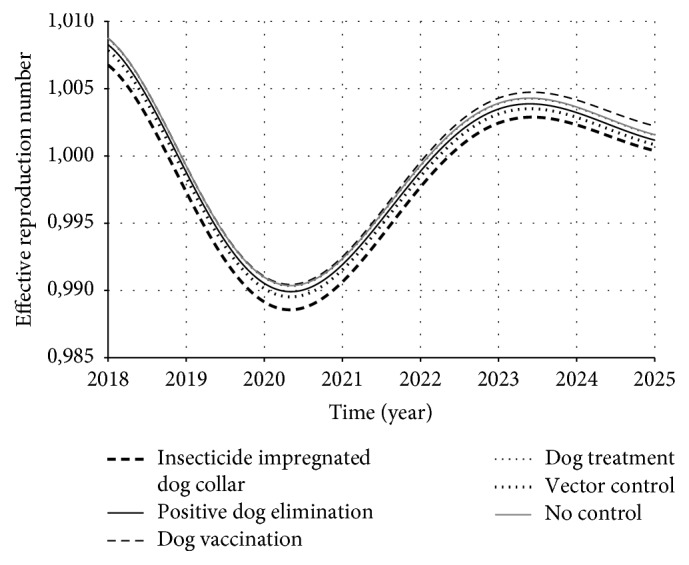
*ℛ*(*t*) dynamics for human population over time. The control strategies were supposed to be introduced from 2018. Observe that insecticide impregnated dog collar was the strategy that most reduced *ℛ*(*t*), followed by vector control and positive dog elimination. Note also that dog treatment curve is overlapped with no-control curve.

(16) Rdit=R0i×xdt+1−εcxdCtRhit=R0i×xht,

where *d* stands for dogs, *h* stands for humans, and the index *i* stands for each of the control strategies. Figures 11 and 12 present the dynamic of *ℛ*_*d*_^*i*^(*t*) and *ℛ*_*h*_^*i*^(*t*), respectively, over time.

Observing both Figures 11 and 12 we see that *ℛ*(*t*) dynamics for dog and human population have similar behavior, but some strategies worked better on dog population than human population (and vice versa). In the case of dog population, the insecticide impregnated dog collar and dog vaccination presented higher reduction of *ℛ*(*t*) than the other strategies. On the other hand, in the case of human population, insecticide impregnated dog collar was the strategy that most reduced *ℛ*(*t*), followed by vector control and positive dog elimination. Those results reflect the fact that humans and dogs play different roles in ZVL chain. Thus, since each strategy acts in a specific point of this chain, they also present different impacts on each population.

## 10. Control Strategies Analysis: The Best Efficacy and Investment Result

At this point of this study, for each control strategy, we estimated the total of saved humans, controlled individuals (dogs or houses), and cost of investment, normalized by human treatment.

In order to make a decision about which control strategy is the most efficient and cost-effective, a simple criterion was adopted. This criterion analyzed the amount of controlled individuals necessary to avoid one human to become clinically ill. In the same way, it is also possible to analyze how much the investment for each strategy to have one saved human is.

For each strategy we calculated the ratio total of controlled individuals/total of saved humans (17) and total cost/total of saved humans (18).

(17) $$\begin{array}{c} I_{i}\left(\;t_{f}\;\right)=\frac{T_{i}\left(t_{f}\right)}{T_{saved}^{i}\left(t_{f}\right)}, \end{array}$$

where *ℑ*_*i*_ means the ratio of total controlled individuals/total saved humans, *T*_*i*_ is the total of controlled elements, *𝒯*_saved_^*i*^ means the total of saved humans, *i* stands for the respective control strategy, and *t*_*f*_ is the final time.

(18) $$\begin{array}{c} C_{i}\left(\;t_{f}\;\right)=I_{i}\left(\;t_{f}\;\right)\times C_{i}, \end{array}$$

where *𝒞*_*i*_ is the cost of the strategy per individual.

**Figure 13:**
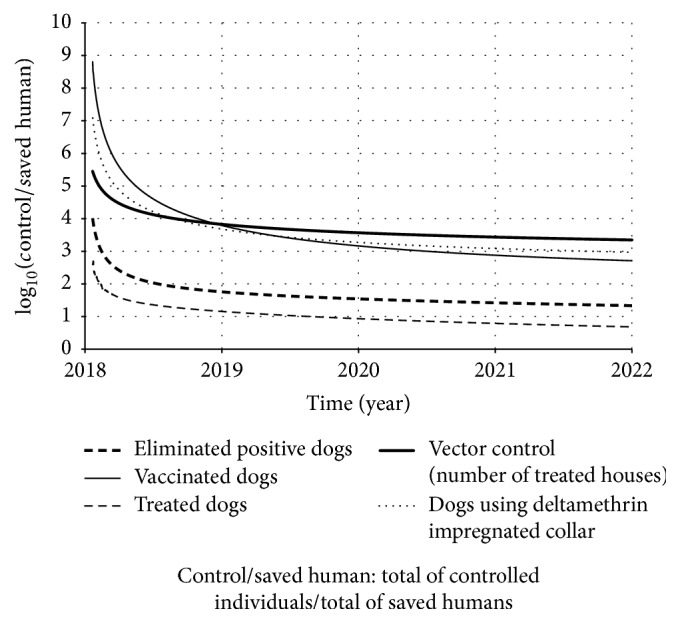
Result of the simulations of expression (17) over time, according to each strategy.

**Figure 14:**
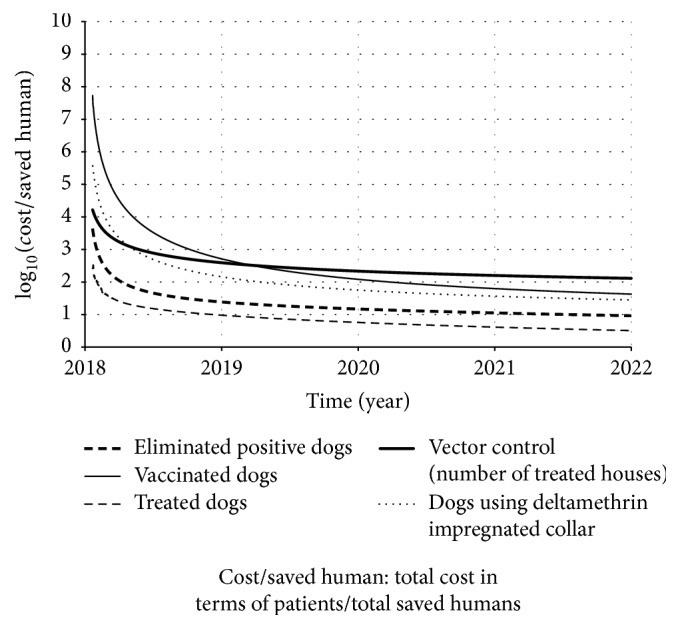
Result of the simulations of expression (18) over time, according to each strategy.

Figures 13 and 14 present the result of those ratios over time.

From Figure 13, we observed that insecticide impregnated dog collar, dog vaccination, and sandfly population control were the strategies that require more elements to be controlled (in the case of collar or vaccination, we refer to dogs; in the case of sandfly population control, we refer to houses). In other words, those three strategies need to be applied in more individuals (or houses) in order to avoid one human being infected by LVZ. Our argument is based on the number needed to be treated (NNT) conception, which means how many individuals need to be controlled in order to avoid one infected individual.

On the other hand, as more elements are controlled, the total cost becomes higher. Thus, the cost of those three strategies was also the one which required more investment among the control strategies studied (Figure 14). However, among those three strategies, we noted that the insecticide impregnated dog collar prevails as the strategy that most saved humans (Figure 8). And, observing Figure 14, we see that the insecticide impregnated dog collar also prevails as the less expensive strategy (per saved human) among those three (followed by dog vaccine and sandfly population control). Therefore, according to our model, the insecticide impregnated dog collar should be the first-choice control strategy, if used isolated.

## 11. Discussion

In this work, we analyzed the impact and cost-effectiveness of five control strategies considering basic mathematical model, published by Burattini et al. [10] and Ribas et al. [11]. Here, we not only updated most of parameters but also developed a study of those strategies regarding reported human cases prediction, *ℛ*(*t*) dynamics, and investment to control one individual (dog or house) in terms of human patient cost.

According to our modeling of each strategy, it became clearer to understand how each one works in the prevention of infection in humans. First of all, remember that dogs are the main source of infection and sandfly bite is the main transmission way. Therefore, positive dog elimination strategy reduces the source of infection available by instantaneous remotion and avoids more noninfected sandflies acquiring the parasites. Dog treatment strategy also works reducing the source of infection, but without elimination. However, treating the dog does not necessarily eliminate the parasite from dog's organism. Dog vaccination does not eliminate the source of infection, but it protects the remaining susceptible dogs to become infected. Thus, there is the reduction of infected dog by natural elimination. The use of insecticide impregnated dog collar (if used by all dogs) works by protecting the susceptible ones (similar to the vaccine activity) and isolating the source of infection (similar to positive dog elimination). Finally, the sandfly population control aims at reducing the chance of disease transmission by intensifying the cycle of life of the mosquito. As a consequence, if the replacing of mosquito is accelerated, there is no time enough to mature the parasite inside the sandfly. In other words, the cycle of life of mosquito is not long enough to support the incubation period.

The comprehension of how each strategy works on the epidemiologic chain allows us to better understand the results of this study. For instance, since the dog treatment has shown a probability of parasitemia elimination around 84.6% [45], some clinically ill dogs would remain as source of infection; besides they become visually healthy. Since it is more probable for noninfected sandfly to acquire the parasite from a latent rather than a clinically ill dog [33], the dog treatment allows some dogs to remain as source of infection. From a public health point of view, this is epidemiologically undesirable, because there is the probability of increasing the proportion of latent dogs. This explains why the curve regarding dog treatment was considered the less efficient one and, in some cases, was overlapped with no-control curve.

On the other hand, we observed some differences among the impacts of control strategies on human and dog populations. All control strategies are applied on dog or sandfly population and, therefore, the impact on those populations reflects on human population. However, the consequences on human population are not immediate. This helps us to understand the fact that the impact on dog population is more intense and faster than on human population.

Although the consequences on each population are different, in both the use of insecticide impregnated dog collar presented the most positive impact in terms of disease control. This strategy not only reduces the frequency of contact between dogs and sandflies but also reduces the infective sandfly population. As a consequence, the probability of a susceptible human acquiring the infection is also decreased.

Following the insecticide impregnated dog collar, we found different strategies depending on the population. If we observe the dog population, dog vaccination presented a good result. Classically, the vaccination is well known as a preventive strategy, as it removes the susceptible individuals to a vaccinated category, in which it is immune to infection. But, if we analyze the human population, we found that sandfly population control and positive dog elimination were the strategies that presented good results. First, we have to remember that humans are not source of infection and, therefore, the objective for this population is to decrease the force of infection. The force of infection is mathematically defined as *λ*_*i*_ = *b*_*i*_ × *a*_*i*_ × *m*_*i*_ × *s*_3_(*t*) [48], where *i* = *h*, *d*. According to our results, to reduce the intensity of source of infection, it is necessary to control *s*_3_(*t*). Basically, focusing on human population, the reduction of *s*_3_(*t*) is most efficient if we consider the sandfly population control (as the sandfly life cycle becomes shorter) and positive dog elimination (we obtain the immediate elimination of source of infection). In the case of human population, dog vaccination presented a low impact, since the vaccination of dog does not immediately remove the infected dog. In this case, those dogs would be naturally eliminated and they would be able to continue playing as source of infection.

Our model provided us with those important results, but it is also necessary to consider real-world restrictions. Here we may simplify those restrictions explaining that they include economical, material, and human resources. Regarding visceral leishmaniasis, there are few works that economically evaluated the preventive control strategies [9]. There are studies that presented an economical analysis approaching treatment and diagnosis of human cases [9, 49, 50] and one of the conclusions pointed out was that investing on preventive activities is beneficially economical [49, 51, 52]. In our study, we not only elaborated a cost-effectiveness evaluation but also observed it dynamically. However, even though our study was based on simple analysis (e.g., we did not include disability adjusted life years or potentially productive years of life lost), our results are very important to fulfill a gap between epidemiological and economical analysis.

In our study, we estimated the total of saved humans and of controlled individuals (dogs or houses) over time and we found that insecticide impregnated dog collar, dog vaccination, and sandfly population control were the ones that saved more humans. On the other hand, they required more individuals to be controlled and, as a consequence, they required more investments too (Figures 9 and 10). Observing Figure 10, dog treatment was the less expensive strategy. If we strongly impose the financial resource as restriction (or if our priority is to save financial resources), we should choose treating dog as control strategy. However, we have already known that this strategy presented low effect to control this disease. Therefore, we need to find equilibrium between the control efficacy and cost-effectiveness.

Figure 10 pointed out that the sandfly population control, insecticide impregnated dog collar, and dog vaccination are the most expensive strategies, if we consider the total cost. This is in agreement with the results described by Camargo-Neves [31] in her field study at Araçatuba municipality. This result can be biologically explained as follows. First, a dog lives for a time longer than a sandfly and the sandfly/dog ratio is higher than the sandfly/house ratio. Thus, if only sandfly population control operates as control strategy, we would have to keep sandfly elimination rate *ξ*_*c*_ until the density of latent and clinically ill dogs reduction reflects on the reduction of reported human cases rate. In other words, while sandflies would be eliminated, it would also be necessary to wait for dog's natural death. This fact would result in continued remotion of sandflies, in which it would generate a fixed cost rate. Still, if the latent and clinically ill dogs are reduced by elimination, the impact on prevalence would be more intense, since the positive dog elimination is an immediate way to reduce source of infection. In the course of time, if the positive dog elimination is kept constant, the number of eliminated dogs tends to decrease. However, we should remember that sandfly population control may be an interesting option in terms of cost-effectiveness, depending on social and economical aspects of the area [52].

Although we have not found any economical study regarding dog vaccination, Lee et al. [53] presented an economical analysis for human vaccine. Although the authors considered some disease dynamics hypothesis different from the one from Brazil, it was demonstrated by computational simulations that vaccination can be cost-effective. However, more studies are necessary to understand the real impact of visceral leishmaniasis vaccination as control strategy [6, 44, 53].

In order to make a correct decision, we need to find a relationship between the total of investment on control strategy and the total of saved humans. From this relation, we can understand how expensive it was to prevent human from becoming clinically ill (see expression (18)). Observing Figure 13, we note that, in most of the simulated period, sandfly control population was the strategy that required more elements (houses) to be controlled per saved human. On the other hand, this is the opposite of dog treatment, in which we had fewer elements (dogs) to be treated per saved human. However, despite the fact that dog treatment required fewer elements per saved human, it also resulted in lowest impact among all considered strategies.

But if the controlled elements/saved human ratio is changed to control strategy cost/saved human ratio, we obtain a new approach. According to Figure 14, among those three most efficient strategies (insecticide impregnated dog collar, dog vaccination, and sandfly population control), the insecticide impregnated dog collar was the strategy that showed the best relation of epidemiological control with cost-effectiveness. This is in agreement with a field study developed by Camargo-Neves et al. [47] at Andradina municipality (São Paulo State, Brazil), in which the impacts of insecticide impregnated dog collar against sandfly population control were compared and it was found that the use of dog collar was economically more convenient. In this case, the dog collar is able to repel the sandfly, reducing the contact between dog and sandfly. Also, it avoids both the dog to become infected and the noninfected sandfly to acquire the parasite.

## 12. Conclusion

In this work, we presented an evaluation of ZVL control strategies, considering epidemiological control impact and cost-effectiveness as analysis criteria. Our results pointed out that focusing the control activities on source of infection and on sandfly population is the way to reach the optimal control and that is why insecticide impregnated dog collar was considered the most efficient and cost-effective among the control strategies. However, since human and dog populations play different roles in this epidemiological chain, choosing criteria on the best control strategy is different. Furthermore, as each control strategy works in different points of disease maintenance and transmission, there is the possibility of improving the disease control results by operating more than one strategy simultaneously. The combination of two or more control strategies is in our upcoming works.

## Figures and Tables

**Table 1:** Parameters adopted in our model. The indexes *h*, *d,* and *s* stand for humans, dogs, and sandflies, respectively.

Parameter	Meaning	Value	Dimension	Source
*μ*_*h*_	Natural mortality rate	3.67 × 10^−5^	1/day	Brazilian Institute of Geography and Statistics, Brazil [19]
*α*_*h*_	Kalazar specific lethality	6.31 × 10^−3^	1/day	World Health Organization [20]
*a*_*h*_	Average daily bitten humans rate	2.00 × 10^−1^	Human/(sandfly × day)	Epidemiological Surveillance Direction, Santa Catarina State, Brazil [21]
*m*_*h*_(*t*)	Vector density per host (time-dependent)	*Variable*	Sandfly/human	Fitted
*w*_*hc*_	Human : house ratio	3	Human/house	Brazilian Institute of Geography and Statistics, Brazil [22]
*b*_*h*_	Proportion of infective bites	1.00 × 10^−2^	Dimensionless	Molineaux and Gramiccia [23]
*r*_*h*_	Spontaneous recovery rate	5.48 × 10^−4^	1/day	Badaro et al. [24]
*γ*_*h*_	Loss of immunity rate	5.48 × 10^−4^	1/day	Kault and Marsh [25]
*δ*_*h*_	Latent recovery rate	1.10 × 10^−2^	1/day	Badaro et al. [24]
*φ*_*h*_	Inverse of incubation period	4.00 × 10^−4^	1/day	Pearson and Souza [26]
*σ*_*h*_	Recovery rate to immunes	2.50 × 10^−3^	1/day	Ministry of Health, Brazil [17]
*μ*_*d*_	Natural mortality rate	2.28 × 10^−4^	1/day	Selman et al. [27]
*α*_*d*_	Kalazar specific lethality	1.81 × 10^−3^	1/day	Lanotte et al. [28]
*a*_*d*_	Average daily bitten dogs rate	2.00 × 10^−1^	Dog/(sandfly × day)	Epidemiological Surveillance Direction, Santa Catarina State, Brazil [21]
*w*_*dh*_	Human : dog ratio for Araçatuba/SP city	10/1.8	Human/dog	Andrade et al. [29]
*m*_*d*_(*t*)	Vector density per host	*w*_*dh*_ × *m*_*h*_(*t*)	Sandfly/dog	—
*φ*_*d*_	Inverse of incubation period	3.78 × 10^−4^	1/day	Greene [30]
*b*_*d*_	Proportion of infective bites	1.00 × 10^−2^	Dimensionless	Molineaux and Gramiccia [23]
*r*_*d*_	Spontaneous recovery rate	2.74 × 10^−4^	1/day	Lanotte et al. [28]
*γ*_*d*_	Loss of immunity rate (recovery to susceptible)	2.74 × 10^−3^	1/day	Kault and Marsh [25]
*σ*_*d*_	Recovery rate from clinically ill to immunes	9.04 × 10^−4^	1/day	Lanotte et al. [28]

**Table 2:** Parameters adopted in our model. The indexes *h*, *d,* and *s* stand for humans, dogs, and sandflies, respectively (continuation of Table 1).

Parameter	Meaning	Value	Dimension	Source
*δ*_*d*_	Latent recovery rate	8.22 × 10^−3^	1/day	Lanotte et al. [28]
*ξ*_*d*_	Dog elimination rate	3.36 × 10^−4^	1/day	Camargo-Neves [31]
*μ*_*s*_	Natural mortality rate	5.00 × 10^−2^	1/day	Ministry of Health, Brazil [17]
*τ*	Extrinsic incubation period	7	Day	Neva and Sacks [32]
*a*_*S*_	Average daily biting rate (on dogs)	2.00 × 10^−1^	1/day	Estimated as Epidemiological Surveillance Direction, Santa Catarina State, Brazil [21]
*c*_*l*_	Probability of latent dog to infect the sandfly	0.385	Dimensionless	Laurenti et al. [33]
*c*_*y*_	Probability of clinically ill dog to infect the sandfly	0.247	Dimensionless	Laurenti et al. [33]

**Table 3:** Human and dog demographic data from Araçatuba municipality and estimated human reported cases.

Year	Human reported cases per year (CES-SP) [36]	Araçatuba's human population size (BIGS) [22]	Average of normalized human reported cases per day	Estimated dog population according to Andrade et al. [29]	Estimated number of houses (BIGS) [22]
1999	15	169303	2.43 × 10^−7^	30475	56434
2000	12	170296	1.93 × 10^−7^	30653	56765
2001	29	171289	4.64 × 10^−7^	30832	57096
2002	52	172768	8.25 × 10^−7^	31098	57589
2003	40	174399	6.28 × 10^−7^	31392	58133
2004	41	177823	6.32 × 10^−7^	32008	59274
2005	16	179717	2.44 × 10^−7^	32349	59906
2006	20	181598	3.02 × 10^−7^	32688	60533
2007	42	181371	6.34 × 10^−7^	32647	60457
2008	27	181143	4.08 × 10^−7^	32606	60381
2009	15	182204	2.26 × 10^−7^	32797	60735
2010	4	182365	6.01 × 10^−8^	32826	60788
2011	5	182526	7.51 × 10^−8^	32855	60842
2012	6	183441	8.96 × 10^−8^	33019	61147
2013	3	190536	4.31 × 10^−8^	34296	63512
2014	12	191662	1.72 × 10^−7^	34499	63887
2015	4	192757	5.69 × 10^−8^	34696	64252

**Table 4:** Parameter values for (7) and their biological meaning.

Parameter	Meaning	Value	Dimension	Source
*m*_*h*0_	Vector density per host (baseline value)	0.75	Sandfly/human	Fitted
*A*	Vector density per host	3.4	Sandfly/human	Fitted
*B*	Vector density per host	8.3	Sandfly/human	Fitted
*L*	Linear constant	3.0	Dimensionless	Fitted
*K*_1_	Proportionality constant	3.5 × 365	Day	Fitted
*T*	Sandfly population dynamics period	5.5 × 365	Day	Fitted

**Table 5:** Additional parameters adopted for evaluation of deltamethrin 4% impregnated dog collar.

Parameter	Meaning	Value	Dimension	Source
*θ*_*d*_	Rate of dogs using collar	Variable	1/day	—
*u*_*c*_	Inverse of activity period of collar	6.70 × 10^−3^	1/day	Scalibor ® [40]
*ζ*_*c*_	Loss of insecticide impregnated collar	6.00 × 10^−3^	1/day	Reithinger et al. [41]
*ε*_*c*_	Decrease of biting rate due to insecticide impregnated collar	8.00 × 10^−1^	Dimensionless	Halbig et al. [42]

**Table 6:** Additional parameters adopted for evaluation of dog vaccination.

Parameter	Meaning	Value	Dimension	Source
*υ*_*d*_	Leishmaniasis vaccination rate	Variable	1/day	—
*p*_*d*_	Loss of immunity rate (Leishmune® vaccination)	2.74 × 10^−3^	1/day	Moreira [43]
*ε*_*v*_	Efficacy of ZVL vaccination	0.964	Dimensionless	Fernandes et al. [44]

**Table 7:** Additional parameters adopted for evaluation of dog treatment.

Parameter	Meaning	Value	Dimension	Source
*ω*_*d*_	Dog treatment rate	Variable	1/day	—
*c*_*k*_	Proportion of clinically recovered dogs but that are still infected	0.154	Dimensionless	Miró et al. [45]
*ψ*_*d*_	Proportion of dogs that receive the complete treatment	1	Dimensionless	Assumed

**Table 8:** Summary of average costs for strategy controls and for human patient treatment.

	Meaning	Cost	Cost dimension	Source	Normalized cost (in terms of patient cost)	Normalized cost dimension	Control rate	Control rate dimension
*ξ*′_*d*_	Elimination of positive dog	170.71	USD/dog	Estimated as Camargo-Neves [31]	0.43	Patient/dog	3.69 × 10^−6^	1/day
*θ*_*d*_	Deltamethrin 4% impregnated dog collar	12.00	USD/dog	Estimated as Camargo-Neves et al. [47]	0.03	Patient/dog	5.25 × 10^−5^	1/day
*ω*_*d*_	Dog treatment with allopurinol and meglumine antimoniate	265.76	USD/dog	Estimated as Miró et al. [45]	0.67	Patient/dog	2.37 × 10^−6^	1/day
*υ*_*d*_	Vaccine	33.00	USD/dog	F. F. Gonzales (Personel communication, 2016)	0.08	Patient/dog	1.91 × 10^−5^	1/day
*ξ*_*c*_	Sandfly population control	23.24	USD × sandfly/(house)^2^	Estimated as Camargo-Neves [31]	0.06	Patient × sandfly/(house)^2^	1.46 × 10^−5^	House/(sandfly × day)
—	Human patient treatment	397.25	USD/patient	Estimated as Akhavan [46]	1.00	Patient/patient	—	—

**Table 9:** Summary of the expressions for total of controlled elements and for total cost. The initial values of the time interval and the ending are represented by *t*_0_ and *t*_*f*_, respectively. *N*_*d*_ = 34889 is the estimated dog population and *H* = 64609 is the estimated total of houses, both for Araçatuba municipality in 2016.

	Meaning	Total of controlled elements (*T*_*i*_)^†^	Normalized cost^*∗*^ (*𝒞*_*i*_)^†^	Normalized cost dimension	Total cost^*∗*^ (*C*_*i*_^*T*^)^†^
*ξ*′_*d*_	Elimination of positive dog	Ndξ′d∫t0tfldt+ydt+zdtdtkk	0.43	Patient/dog	*T*_*ξ*′_*d*__ × *𝒞*_*ξ*′_*d*__
*θ*_*d*_	Deltamethrin 4% impregnated dog collar	*N*_*d*_ *θ*_*d*_∫_*t*_0__^*t*_*f*_^(*x*_*d*_(*t*) + *l*_*d*_(*t*) + *y*_*d*_(*t*) + *z*_*d*_(*t*))*dt*	0.03	Patient/dog	*T*_*θ*_*d*__ × *𝒞*_*θ*_*d*__
*ω*_*d*_	Dog treatment with allopurinol and meglumine antimoniate	*N*_*d*_ *ω*_*d*_∫_*t*_0__^*t*_*f*_^*y*_*d*_(*t*)*dt*	0.67	Patient/dog	*T*_*ω*_*d*__ × *𝒞*_*ω*_*d*__
*υ*_*d*_	Vaccine	*N*_*d*_ *υ*_*d*_∫_*t*_0__^*t*_*f*_^(*x*_*d*_(*t*) + *l*_*d*_(*t*) + *y*_*d*_(*t*) + *z*_*d*_(*t*))*dt*	0.08	Patient/dog	*T*_*υ*_*d*__ × *𝒞*_*υ*_*d*__
*ξ*_*c*_	Vector control	Hξc∫t0tfdtkk	0.06	Patient/house	*T*_*ξ*_*c*__ × *𝒞*_*ξ*_*c*__

^*∗*^In terms of patient cost.

^†^The index *i* stands for the respective control strategy.

**Table 10:** Summary of the expressions for total of controlled elements and for total cost. The initial values of the time interval and the ending are represented by *t*_0_ and *t*_*f*_, respectively. *N*_*d*_ = 34889 is the estimated dog population and *N*_houses_ = 64609 is the estimated total of houses, both for Araçatuba municipality in 2016.

	Meaning	*ℛ*_0_ expression
*ξ*′_*d*_	Elimination of positive dog	ℛ0ξ′d=wdhmh0bdadase-μsτclσd+αd+μd+ξd+ξ′d+cyφdrd+δd+φd+μd+ξd+ξ′dσd+αd+μd+ξd+ξ′dμsιιι

*θ*_*d*_	Deltamethrin 4% impregnated dog collar	ℛ0θd=wdhmh0adasbde-μsτa1a3a1+a2+θda3+a2+θdμs×P1+P2,ιιι
		where
		*P*_1_ = *c*_*l*_*a*_3_(*a*_1_ + *a*_2_ + (1 − *ε*_*c*_)*θ*_*d*_)(*a*_2_ + *a*_3_ + *θ*_*d*_)
		*P*_2_ = *c*_*l*_*φ*_*d*_(*a*_1_*a*_3_ + (*a*_2_ + (1 − *ε*_*c*_)*θ*_*d*_))(*a*_1_ + *a*_2_ + *a*_3_ + *θ*_*d*_)
		*a*_1_ = *μ*_*d*_ + *r*_*d*_ + *δ*_*d*_ + *φ*_*d*_ + *ξ*_*d*_
		*a*_2_ = *ζ*_*c*_ + *u*_*c*_
		*a*_3_ = *μ*_*d*_ + *α*_*d*_ + *σ*_*d*_ + *ξ*_*d*_

*ω*_*d*_	Dog treatment with allopurinol and meglumine antimoniate	ℛ0ωd=wdhmh0adasbde-μsτclσd+αd+μd+ξd+ψdωd+cyφdrd+δd+φd+μd+ξdσd+αd+μd+ξd+ψdωdμsιιι

*υ*_*d*_^*∗*^	Vaccine	ℛ0υd=wdhmh0adasbde-μsτclσd+αd+μd+ξd+cyφdrd+δd+φd+μd+ξdσd+αd+μd+ξdμsιιι

*ξ*_*c*_	Vector control	ℛ0ξc=wdhmh0adasbde-μs+ξcwhcmh0τclσd+αd+μd+ξd+cyφdrd+δd+φd+μd+ξdσd+αd+μd+ξdμs+ξcwhcmh0ιιι

^*∗*^Note that *ℛ*_0_^*υ*_*d*_^ does not depend on *υ*_*d*_.

## References

[1] Duthie M. S., Raman V. S., Piazza F. M., Reed S. G. (2012). The development and clinical evaluation of second-generation leishmaniasis vaccines. *Vaccine*.

[2] Killick-Kendrick R. (2010). Education is key to controlling visceral leishmaniasis. *Bulletin of the World Health Organization*.

[3] Pan American Health Organization (2001). *Zoonoses and Communicable Diseases Common to Man and Animals*.

[4] World Health Organization Neglected tropical disease (NTD) research. http://www.who.int/tdr/research/ntd/en/.

[5] Palatnik-De-Sousa C. B., Day M. J. (2011). One Health: the global challenge of epidemic and endemic leishmaniasis. *Parasites and Vectors*.

[6] Maia-Elkhoury A. N. S., Alves W. A., De Sousa-Gomes M. L., De Sena J. M., Luna E. A. (2008). Visceral leishmaniasis in Brazil: trends and challenges. *Cadernos de Saúde Pública*.

[7] Brazilian Institute of Geography and Statistic. Brazil (BIGS) *Health Economics. A Macroeconomic Perspective 2000—2005*.

[8] Andrade E. I., Acúrcio F. d., Cherchiglia M. L. (2007). Pesquisa e produção científica em economia da saúde no Brasil. *Revista de Administração Pública*.

[9] Marinho D. S., Casas C. N., Pereira C. C., Leite I. C., Lee B. Y. (2015). Health economic evaluations of visceral leishmaniasis treatments: a systematic review. *PLoS Neglected Tropical Diseases*.

[10] Burattini M. N., Coutinho F. A. B., Lopez L. F., Massad E. (1998). Modelling the dynamic of leishmaniasis considering human, animal host and vector population. *Journal of Biological Systems*.

[11] Ribas L. M., Zaher V. L., Shimozako H. J., Massad E. (2013). Estimating the optimal control of zoonotic visceral leishmaniasis by the use of a mathematical model. *The Scientific World Journal*.

[12] Shimozako H. J., Wu J., Massad E. (2017). Mathematical modelling for Zoonotic Visceral Leishmaniasis dynamics: a new analysis considering updated parameters and notified human Brazilian data. *Infectious Disease Modelling*.

[13] Zhao S., Kuang Y., Wu C.-H., Ben-Arieh D., Ramalho-Ortigao M., Bi K. (2016). Zoonotic visceral leishmaniasis transmission: modeling, backward bifurcation, and optimal control. *Journal of Mathematical Biology*.

[14] Ministry of Health. Brazil Human cases of Visceral Leishmaniasis reporting. http://portalsinan.saude.gov.br/dados-epidemiologicos-sinan.

[15] Vieira J. B., Coelho G. E. (1998). Visceral leishmaniasis or kala-azar: the epidemiological and control aspects. *Revista da Sociedade Brasileira de Medicina Tropical*.

[16] Dye C. (1996). The logic of visceral leishmaniasis control. *American Journal of Tropical Medicine and Hygiene*.

[17] Ministry of Health. Brazil *Guideline of Surveillance and Control of Visceral Leishmaniasis*.

[18] Tesh R. B. (1995). Control of zoonotic visceral leishmaniasis: is it time to change strategies?. *American Journal of Tropical Medicine and Hygiene*.

[19] Brazilian Institute of Geography and Statistics. Brazil (BIGS) In 2012, life expectancy at birth was 74.6 years. http://saladeimprensa.ibge.gov.br/noticias?view=noticia&id=1&busca=1&idnoticia=2528.

[20] World Health Organization Visceral leishmaniasis. http://www.who.int/leishmaniasis/visceral_leishmaniasis/en.

[21] Epidemiological Surveillance Direction; Santa Catarina State; Brazil Guidance manual for training of entomology laboratory technicians. http://www.dive.sc.gov.br/conteudos/zoonoses/capacitacao/guia-orientacao-treinamento-de-tecnicos.pdf.

[22] Brazilian Institute of Geography and Statistics; Brazil (BIGS) São Paulo, Araçatuba. http://cidades.ibge.gov.br/xtras/perfil.php?codmun=350280.

[23] Molineaux L., Gramiccia G. (1980). *The Garki Project*.

[24] Badaro R., Jones T. C., Carvalho E. M. (1986). New perspectives on a subclinical form of visceral leishmaniasis. *Journal of Infectious Diseases*.

[25] Kault D. A., Marsh L. M. (1991). Modeling AIDS as a function of other sexually transmitted disease. *Mathematical Biosciences*.

[26] Pearson R. D., Souza A. Q., Mandell G. L., Douglas-Junior R. G., Bennett J. E. (1990). Leishmania species: visceral (kala-azar), cutaneous and mucosal leishmaniasis. *Principles and Practice of Infectious Diseases*.

[27] Selman C., Nussey D. H., Monaghan P. (2013). Ageing: it's a dog's life. *Current Biology*.

[28] Lanotte G., Rioux J. A., Perieres J., Vollhardt Y. (1979). Ecology of leishmaniasis in the south of France. 10. Developmental stages and clinical characterization of canine leishmaniasis in relation to epidemiology. (author's translation). *Annales de Parasitologie Humaine et Comparee*.

[29] Andrade A. M., Queiroz L. H., Perri S. H. V., Nunes C. M. (2008). A descriptive profi le of the canine population in Araçatuba, São Paulo State, Brazil, from 1994 to 2004. *Cadernos de Saude Publica*.

[30] Greene C. E. (2011). *Infectious Diseases of the Dog and Cat*.

[31] Camargo-Neves V. L. F. (2004). *Epidemiologic aspects and evaluation of the control methods American visceral leishmaniasis in São Paulo State, Brazil [Ph.D. thesis]*.

[32] Neva F., Sacks D., Warren K. S., Mahmoud A. A. F. (1990). Leishmaniasis. *Tropical and Geographical Medicine*.

[33] Laurenti M. D., Rossi C. N., Matta V. L. R. D. (2013). Asymptomatic dogs are highly competent to *transmit Leishmania* (*Leishmania*) *infantum chagasi* to the natural vector. *Veterinary Parasitology*.

[34] Day M. J., Breitschwerdt E., Cleaveland S. (2012). Surveillance of zoonotic infectious disease transmitted by small companion animals. *Emerging Infectious Diseases*.

[35] Maia-Elkhoury A. N. S., Carmo E. H., Sousa-Gomes M. L., Mota E. (2007). Analysis of visceral leishmaniasis reports by the capture-recapture method. *Revista de Saude Publica*.

[36] Centre of Epidemiological Surveillance of São Paulo State (CES-SP); Brazil Visceral Leishmaniasis reported data. http://www.cve.saude.sp.gov.br/htm/cve_leishvis.html.

[37] de Oliveira G. M. G., Figueiró Filho E. A., Andrade G. M. C., de Araújo L. A., de Oliveira M. L. G., da Cunha R. V. (2010). Survey of phlebotomine sand flies (Diptera: Psychodidae: Phlebotominae) in Três Lagoas Municipality, Mato Grosso do Sul State, Brazil, an area of intense transmission of American visceral leishmaniasis. *Revista Pan-Amazônica de Saúde*.

[38] van den Driessche P., Watmough J. (2002). Reproduction numbers and sub-threshold endemic equilibria for compartmental models of disease transmission. *Mathematical Biosciences*.

[39] Massad E., Coutinho F. A. B., Lopez L. F., Da Silva D. R. (2011). Modeling the impact of global warming on vector-borne infections. *Physics of Life Reviews*.

[40] Scalibor® website http://www.medicanimal.com/Scalibor-Collar/p/I0000475.

[41] Reithinger R., Coleman P. G., Alexander B., Vieira E. P., Assis G., Davies C. R. (2004). Are insecticide-impregnated dog collars a feasible alternative to dog culling as a strategy for controlling canine visceral leishmaniasis in Brazil?. *International Journal for Parasitology*.

[42] Halbig P., Hodjati M. H., Mazloumi-Gavgani A. S., Mohite H., Davies C. R. (2000). Further evidence that deltamethrin-impregnated collars protect domestic dogs from sandfly bites. *Medical and Veterinary Entomology*.

[43] Moreira M. L. (2013). *Duração da imunidade vacinal da leishmaniose visceral canina: perfil fenotípico e funcional da atividade fagocítica da anti-Leishmania chagasi [M.S. dissertation]*.

[44] Fernandes C. B., Junior J. T. M., De Jesus C. (2014). Comparison of two commercial vaccines against visceral leishmaniasis in dogs from endemic areas: IgG, and subclasses, parasitism, and parasite transmission by xenodiagnosis. *Vaccine*.

[45] Miró G., Gálvez R., Fraile C., Descalzo M. A., Molina R. (2011). Infectivity to Phlebotomus perniciosus of dogs naturally parasitized with *Leishmania infantum* after different treatments. *Parasites and Vectors*.

[46] Akhavan D. (1996). Análise de custo-efetividade do componente de leishmaniose no projeto de controle de doenças endêmicas no nordeste do Brasil. *Revista de Patologia Tropical*.

[47] Camargo-Neves V. L. F., Rodas L. A. C., Calemes E., Junior C. P., da Silva L. J. Cost effectiveness of deltamethrin impregnated collars (Scalibor®) for the control of visceral leishmaniasis in human and canine populations in Brazil.

[48] Anderson R. M., May R. M. (2010). *Infectious Diseases of Humans: Dynamics and Control*.

[49] Anoopa Sharma D., Bern C., Varghese B. (2006). The economic impact of visceral leishmaniasis on households in Bangladesh. *Tropical Medicine and International Health*.

[50] Boelaert M., Lynen L., Desjeux P., Van Der Stuyft P. (1999). Cost-effectiveness of competing diagnostic-therapeutic strategies for visceral leishmaniasis. *Bulletin of the World Health Organization*.

[51] Adhikari S. R., Supakankunti S. (2010). A cost benefit analysis of elimination of kala-azar in Indian subcontinent: an example of Nepal. *Journal of Vector Borne Diseases*.

[52] Das M., Banjara M., Chowdhury R. (2008). Visceral leishmaniasis on the Indian sub-continent: a multi-centre study of the costs of three interventions for the control of the sandfly vector, *Phlebotomus argentipes*. *Annals of Tropical Medicine and Parasitology*.

[53] Lee B. Y., Bacon K. M., Shah M., Kitchen S. B., Connor D. L., Slayton R. B. (2012). The economic value of a visceral leishmaniasis vaccine in Bihar State, India. *American Journal of Tropical Medicine and Hygiene*.

